# Cardiovascular changes during peanut-induced allergic reactions in human subjects

**DOI:** 10.1016/j.jaci.2020.06.033

**Published:** 2021-02

**Authors:** Monica Ruiz-Garcia, Joan Bartra, Olaya Alvarez, Ashna Lakhani, Shalinee Patel, Alistair Tang, Marcus Sim, Mohamed H. Shamji, Isabel Skypala, E. N. Clare Mills, Alexander R. Lyon, Carl Hayward, Stephen R. Durham, Paul J. Turner, Robert J. Boyle

**Affiliations:** a Section of Inflammation, Repair and Development, National Heart & Lung Institute, Imperial College London, London, United Kingdom; b Hospital Clínic Barcelona, Barcelona, Spain; c Royal Brompton and Harefield Hospitals NHS Foundation Trust, London, United Kingdom; d Division of Infection, Immunity and Respiratory Medicine, School of Biological Sciences, University of Manchester, Manchester, United Kingdom; e Myocardial Function, National Heart & Lung Institute, Imperial College London, London, United Kingdom; f Cardiology Department, Royal Brompton and Harefield Hospitals NHS Foundation Trust, London, United Kingdom; g Barts Health NHS Trust, London, United Kingdom

**Keywords:** Anaphylaxis, cardiac output, cardiovascular, food allergens, intravenous fluids, management, stroke volume, venous return, BP, Blood pressure, CO, Cardiac output, DBPCFC, Double-blind placebo-controlled food challenge, HR, Heart rate, HRV, Heart rate variability, IQR, Interquartile range, OCR, Objective clinical reaction, SV, Stroke volume, VAS, Visual analog scale

## Abstract

**Background:**

Food allergy is the most common cause of anaphylaxis. Changes in posture during acute reactions can trigger fatal outcomes, but the impact of allergic reactions on the cardiovascular system in nonfatal reactions remains poorly understood.

**Objective:**

Our aim was to systematically evaluate changes in cardiovascular function during acute allergic reactions to peanut.

**Methods:**

Participants underwent double-blind placebo-controlled food challenge to peanut as part of a clinical trial. Changes in hemodynamic parameters (heart rate, stroke volume, blood pressure, and peripheral blood flow) and electrocardiogram findings during food challenges were assessed using noninvasive continuous monitoring.

**Results:**

A total of 57 adults (median age 24 years [interquartile range = 20-29]), 53% of whom were female, participated; 22 (39%) had anaphylaxis. Acute reactions were associated with significant changes in stroke volume (mean decrease of 4.2% [95% CI = 0.8-7.6; *P* = .03]), heart rate (mean increase 11.6% [95% CI = 8.4-14.8; *P* < .0001]), and peripheral blood flow (mean increase 19.7% [95% CI = 10.8-28.6; *P* < .0001]), irrespective of reaction severity. These changes were reproduced at a subsequent repeat peanut challenge in 26 participants, and could be reversed with administration of intravenous fluids which resulted in faster resolution of abdominal symptoms.

**Conclusions:**

In this first detailed human study of cardiovascular changes during food-induced allergic reactions, we found evidence for significant fluid redistribution, independent of reaction severity. This provides a sound rationale for optimizing venous return during significant allergic reactions to food. Finally, these data provide a new paradigm for understanding severity in anaphylaxis, in which poor outcomes may occur as a result of a failure in compensatory mechanisms.

## Graphical abstract

**Figure:**
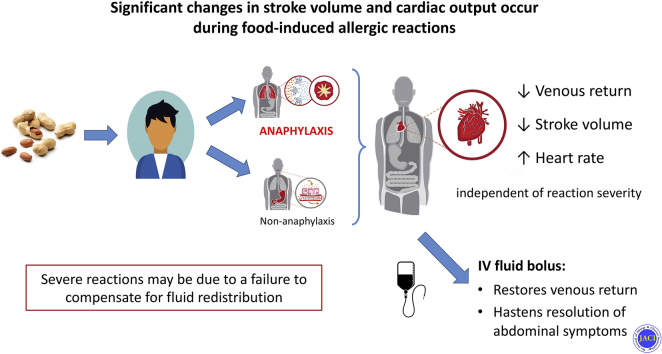

Food allergy is a major public health issue, not only having an impact on affected individuals and their families but also with significant implications for food businesses, educational establishments, and health care systems. It is the most common cause of potentially life-threatening allergic reactions (anaphylaxis), which are an increasing cause of hospitalizations globally.^1^ Anaphylaxis is an IgE-mediated hypersensitivity reaction involving the release of preformed mediators from mast cells and basophils,^2^ many of which have known cardiovascular effects.^2^^,^^3^ However, the pathophysiology of anaphylaxis remains poorly understood,^2^ and severe anaphylaxis is unpredictable and initially, often difficult to distinguish from more mild reactions.^4^ Changes in posture during anaphylaxis have precipitated cardiorespiratory arrest and death,^5^^,^^6^ implying that circulatory compromise is an important feature of some severe reactions.

The management of anaphylaxis has changed little in the past 3 decades, and the evidence base underlying international guidelines is weak, in part because of the difficulty of undertaking randomized controlled trials in this area. To address these knowledge gaps, we systematically evaluated changes in cardiovascular function in adults undergoing acute allergic reactions to peanut under medical supervision.

## Methods

This was an observational study of adults with peanut allergy who were recruited to a randomized crossover study (TRACE Peanut Study), described elsewhere.^7^ The criteria for inclusion were being an adult (aged 18-45 years) with a clinical history and laboratory testing results consistent with IgE-mediated peanut allergy and no known cardiovascular abnormality. Participants underwent initial double-blind, placebo-controlled food challenge (DBPCFC) to peanut to confirm their allergic status, followed by up to 3 further open peanut challenges at 3-month intervals with associated cofactors (sleep deprivation or exercise) or no cofactor (termed *nonintervention challenge*). Cardiovascular assessments were undertaken during the baseline DBPCFC and subsequent nonintervention challenge at the London study site. All participants gave written informed consent (NHS Human Research Authority reference number 15/LO/0286; ClinicalTrials.gov identifier NCT02665793).

### Oral food challenge procedures

The initial DBPCPC was conducted over 2 separate days at least 7 days apart. Subjects received increasing doses, every 30 minutes, of peanut protein (or placebo) concealed in a water-continuous dessert base matrix at doses of 3 μg, 30 μg, 300 μg, 3 mg, 30 mg, 100 mg, 300 mg, and 1000 mg until the criteria were met.^7^ The order for the DBPCFC (ie, placebo or active) was determined by computer randomization. Aside from the technician preparing the challenge material, the members of the study team were blinded as to the challenge assignment. Anaphylaxis was defined according to the National Institute of Allergy and Infectious Disease/Food Allergy and Anaphylaxis Network definition,^8^ but also incorporating laryngeal and isolated lower respiratory symptoms as per the World Allergy Organization recommendations.^9^ Gastrointestinal symptoms were defined as persistent if they lasted more than 30 minutes and/or if there was more than 1 episode of emesis and/or diarrhea (with the episodes occurring at least 20 minutes apart). Reaction severity was assessed according to Sampson.^10^ Epinephrine was administered to treat reactions that presented with significant and objective respiratory and/or cardiovascular symptoms, consistent with local practice in the United Kingdom.^11^ Subsequent challenges were performed by using an identical dosing regime and stopping criteria. Following reaction, subjects rated the severity of their reaction by using a 10-point visual analog scale (VAS) for cutaneous, gastrointestinal, and upper and lower respiratory symptoms.

### Cardiovascular physiology assessments

Participants were monitored continuously by using a validated, US Food and Drug Administration–approved noninvasive method to record stroke volume (SV) through bioreactance and blood pressure (BP) by oscillometry (Nicom, Cheetah Medical, Boston, Mass).^12^^,^^13^ SV and cardiac output (CO) measurements were normalized to each participants’ body surface area. Peripheral skin blood flow was measured by using laser Doppler (moorVMS-LDF laser Doppler, Moor Instruments, Axminster, United Kingdom) over the nape of the neck; measurements were normalized by expressing blood flow as the percentage of maximum blood flow (assessed before challenge by applying heat at 44^o^C for 30 minutes).^14^ To control for movement artifact, patients were kept in a semirecumbent position throughout all challenges.

Electrophysiologic assessments (electrocardiography [ECG] and heart rate [HR] variability [HRV]) were performed by using a 12-lead Holter monitor (SEER 12 Digital ECG Recorder, GE Healthcare, Chicago, Ill), according to international guidelines.^15^ Data were analyzed by using MARS program for ECG data (GE Healthcare). The parameters evaluated included HR, QRS complex, PR, QT, and QTc intervals. HRV is the normal beat-to-beat HRV (RR interval), and it is a measure representing the relative balance between sympathetic and parasympathetic components in the autonomic control of the heart.^16^ HRV was evaluated according to international consensus^15^ by using Kubios HRV software (version 3.0, Kubios, Kuopio, Finland); further details are provided in the Online Repository (available at at www.jacionline.org)***.***

### Other assessments

Blood samples were collected from an intravenous catheter (sited before challenge) at the following time points: baseline, time of objective clinical reaction (OCR), and 30 and 120 minutes after OCR. In the absence of clinical symptoms (eg, placebo visits), samples were drawn 1 hour following the final challenge dose. Samples were processed according to the manufacturers’ instructions and snap-frozen at –80^o^C until analysis.

Total IgE, level of specific IgE to crude peanut extract and recombinant peanut allergen components (recombinant *Arachis hypogaea* 1, 2, 3, 6, 8, and 9), as well as mast cell tryptase level, were measured using by ImmunoCAP (ThermoFisher Scientific, Uppsala, Sweden). Skin prick testing was undertaken according to international guidelines by using ALK lancets and commercial peanut extract (Stallergenes, France) with 1% histamine as a positive control. Plasma epinephrine was determined by using HPLC with electrochemical detection within the Department of Clinical Biochemistry at the Royal Brompton Hospital (accreditation provided by the United Kingdom Accreditation Service).

### Statistical analyses

For the primary analyses, we analyzed 10-minute epochs at baseline (before the first challenge dose) and immediately before OCR (or the equivalent time point for placebo challenges). SV and HR data were averaged for the 30-second measurements (20 in all) taken within each 10-minute epoch before OCR and for the 5-minute epochs following OCR. Peripheral blood flow was expressed as percentage of maximum observed from the onset of any objective symptom through to OCR (or the equivalent time points for nonreactive days), excluding any spikes less than 5 minutes in duration. Data were extracted by members of the study team who had been blinded to the challenge outcome. Data were explored by using Prism software (GraphPad Software, San Diego, Calif) to assess for normality and examine the presence of outliers. Parametric data were analyzed by using the paired *t* test, whereas the Wilcoxon signed rank test and Mann-Whitney *U* test were used for analysis of paired and nonpaired nonparametric data, respectively. For correlation coefficients on nonparametric data, the Spearman rank test was used. *P* values less than .05 were considered statistically significant.

On the basis of the preliminary data relating to 10 participants undergoing peanut DBPCFC, we estimated that a sample size of 50 would provide 80% power at a 2-sided alpha of 0.05 to detect a minimum change of 10 beats per minute for HR, 10 mL/beat for SV, 10 mm Hg for systolic BP and diastolic BP, 10% change in peripheral blood flow, and for electrical changes with a prolongation of 10 ms in QT_c_ and PR intervals and 5 ms for QRS complex.

## Results

**Fig 1:**
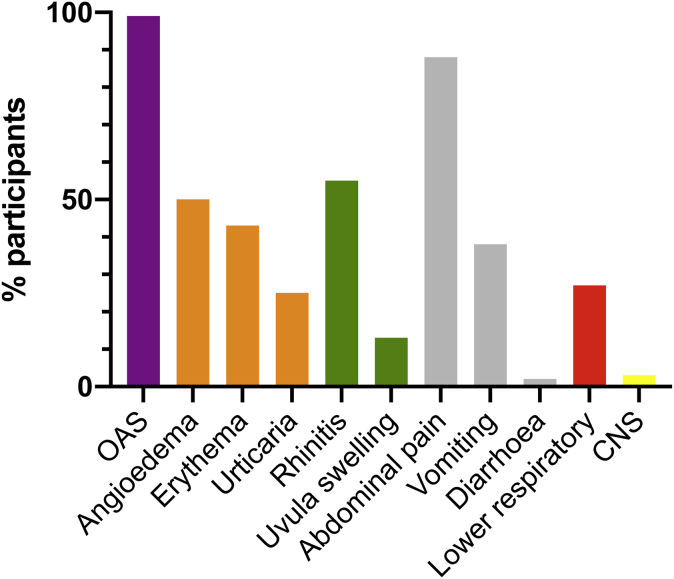
Clinical symptoms observed at food challenge. *OAS*, Oral allergy symptom; *CNS*, change in level of consciousness.

A total of 57 adults underwent initial DBPCFC and reacted with objective symptoms (Table I). Of these 57 individuals, 26 underwent at least 1 further challenge (see Fig E1 [a participant flow diagram] in this article's Online Repository at www.jacionline.org). Oropharyngeal and gastrointestinal symptoms predominated (Fig 1). Reactions were associated with a modest but significant increase in mast cell tryptase level (from a median of 4.3 ng/mL [interquartile range (IQR) = 2.3-5.0 ng/mL]) at baseline to a median of 5.0 ng/mL (IQR = 4.0-5.7 ng/mL) at reaction, which is an increase of 24% (IQR = 7%-46%; *P* < .001) compared with the value after placebo challenge (see Fig E2, *A* in this article's Online Repository at www.jacionline.org). No objective placebo reactions were noted.

**Table I:** Characteristics of the study population and severity of reactions during the study challenges

Characteristic	Initial DBPCFC (n = 57)	Further open challenge (n = 26)
Age at enrollment (y), median (IQR)	24 (20-29)	25 (22-29)
Age at time of diagnosis (y), median (IQR)	2 (1-6)	2 (1-4)
Sex (female), no. (%)	30 (53%)	17 (65%)
Asthma, no. (%)	29 (51%)	14 (54%)
Rhinitis, no. (%)	40 (70%)	20 (77%)
Eczema, no. (%)	27 (47%)	14 (54%)
Total IgE level (kU_A_/L), median (IQR)	221 (107-576)	254 (123-617)
Specific IgE to peanut (kU_A_/L), median (IQR)	10.2 (3.3-31.9)	15.1 (3.4-34.5)
Specific IgE to *rAra h 2* (kU_A_/L), median (IQR)	7.4 (2.0-20.4)	12.2 (2.1-23.4)
SPT wheal size to commercial peanut extract, median (IQR)	11 mm (9-15)	11 mm (9-13)
Sensitized to tree nuts, no. (%)	33 (58%)	17 (65%)
Sensitized to non-nut food, no. (%)	29 (51%)	15 (58%)
Cumulative reaction dose (mg of peanut protein), median (IQR)	133 (33-433)	133 (33-133)
Cardiovascular measures (at baseline), median (IQR)		
HR (beats/min)	69 (63-76)	74 (68-82)
SV (mL/beat/m^2^)	55 (46-60)	49 (43-58)
Systolic bp (mm Hg)	109 (104-119)	110 (104-120)
Diastolic BP (mm Hg)	71 (67-78)	76 (69-80)
Measures of reaction severity		
Severity grade, no. (%)∗		
I	0 (0%)	1 (4%)
II	22 (39%)	13 (50%)
III	10 (18%)	4 (15%)
IV	25 (44%)	9 (35%)
Anaphylaxis	22 (39%)	8 (31%)
Use of adrenaline to treat anaphylaxis	9 (16%)	4 (15%)
Mast cell tryptase level (% increase), median (IQR)	24% (7%-46%)	29 (6%-455)

*rAra h*, Recombinant *Arachis hypogaea*; *SPT*, skin prick text.

∗ Severity grade assigned according to Sampson.^10^

### SV and other physiologic cardiovascular measures

**Fig 2:**
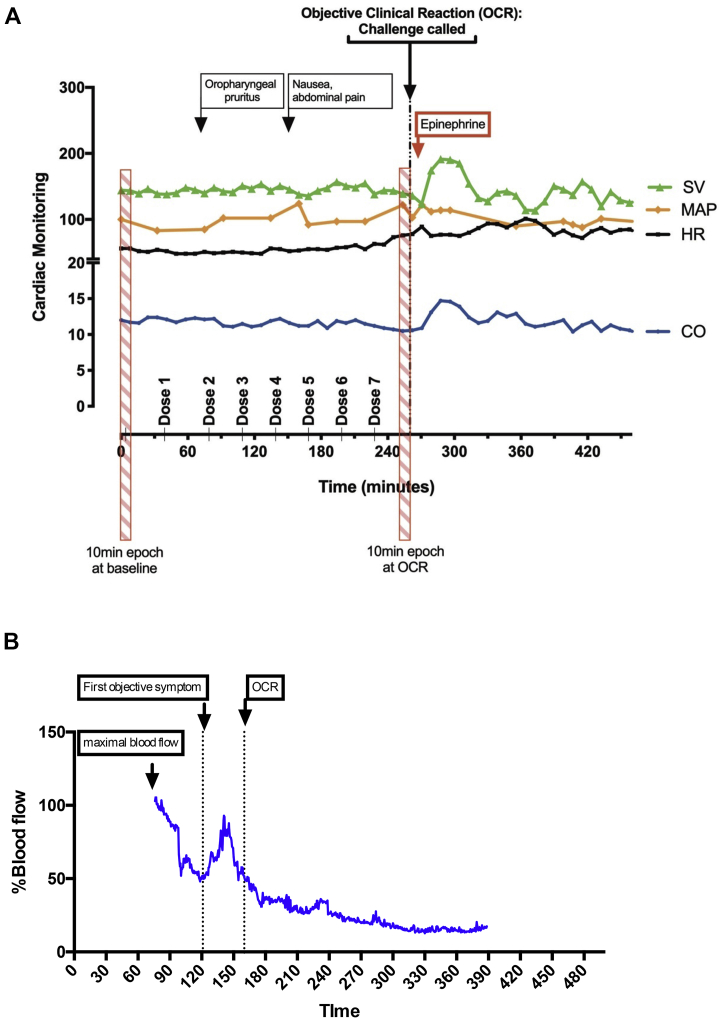
Example traces from continuous noninvasive cardiovascular monitoring during active peanut challenge (**A**) and laser Doppler probe used to monitor cutaneous blood flow (**B**). OCR refers to the time point at which the stopping criteria were fulfilled. *MAP*, Mean arterial BP.

**Fig 3:**
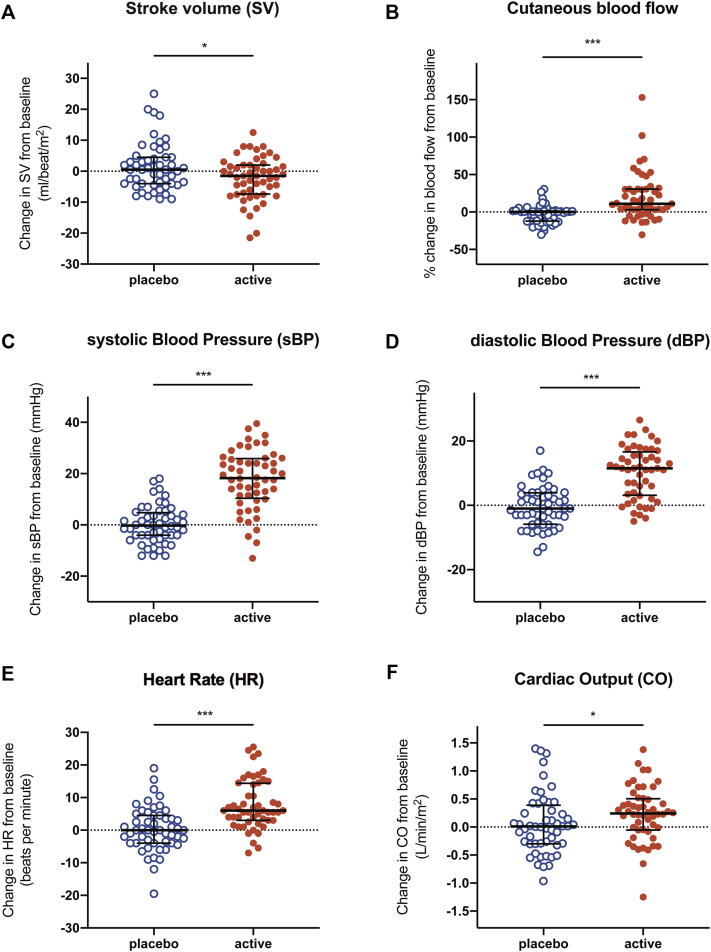
Changes in cardiovascular parameters at time of OCR during peanut-induced allergic reactions at active challenge compared to the placebo challenge: SV (**A**), cutaneous blood flow (measured at the nape of the neck) (**B**), systolic and diastolic BP (**C** and **D**), HR (**E**), and CO (**F**). Line and whiskers indicate medians and IQRs. ∗∗∗∗*P* < .0001; ∗∗∗*P* < .001; ∗*P* < .05; Wilcoxon signed rank test.

**Fig 4:**
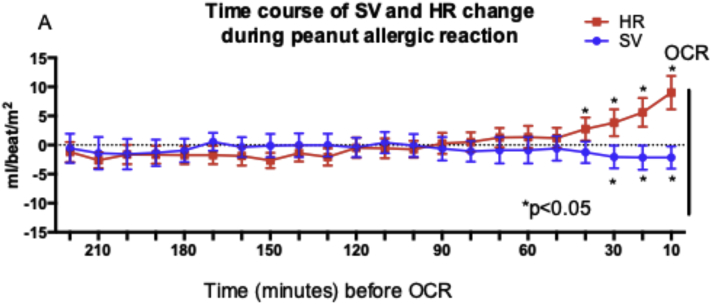
Time course for change in SV and HR before OCR. ∗*P* < .05; Wilcoxon signed rank test.

There was a reproducible decrease in SV at the time of objective symptoms associated with a compensatory increase in HR and BP. SV decreased at OCR (from a mean of 54.6 mL/beat/m^2^ [95% CI = 51.9-57.2] at baseline to mean of 52.3 mL/beat/m^2^ [95% CI = 49.0-55.6 mL/beat/m^2^ at OCR], representing a mean decrease of 4.2% [95% CI = 0.8%-7.6%; *P* = .02]; example trace shown in Fig 2), which was significant compared with the value after placebo challenge (*P* = .03 [Fig 3, *A*]). The decrease in SV was apparent from 30 minutes before OCR (Fig 4). At the same time, there was an increase in peripheral blood flow (a mean increase of 19.7% [95% CI = 10.8%-28.6%; *P* < .0001] [Fig 3, *B*]), and significant increases in systolic BP, diastolic BP, and HR compared with the values for placebo challenge (all *P* < .0001) (Fig 3, *C*-*E*). This resulted in an overall increase in CO at OCR at active challenge (a mean increase of 0.23 L/min/m^2^ [95% CI = 0.10-0.36 L/min/m^2^; *P* = .012 compared with placebo]) (Fig 3, *F*). These changes were not influenced by the order in which subjects were randomized to either active or placebo challenge (see Fig E3 in this article's Online Repository at www.jacionline.org). We did not identify any significant associations between baseline markers of sensitization and the change in any of the cardiovascular parameters evaluated (*P* > .05).

To provide further confidence in these findings, 26 subjects underwent the same assessments at a subsequent open peanut challenge 3 to 12 months later. The baseline characteristics, which are shown in Table I, did not differ from those of the overall cohort. We observed similar changes at OCR: a mean decrease in SV of 7.1% (95% CI = 1.6%-12.6%; *P* = .014 [see Fig E4, *A* in this article's Online Repository at www.jacionline.org]); a mean increase in cutaneous blood flow of 16.3% (95% CI = 3.9%-28.6%; *P* = .03 [see Fig E4, *B*]); and increases in HR, systolic BP, and diastolic BP (*P* < .05, Wilcoxon signed rank test [see Fig E4, *C*-*E*]). We did not, however, observe an increase in CO (*P* = .64 [Fig E4, *F*]).

### Changes in ECG parameters

Cardiac rhythm and HRV were evaluated during both active and placebo challenges in 51 of 57 participants (the analysis could not be completed in 6 subjects because of excessive artifact during at least 1 challenge occasion). Aside from the increase in HR that was observed with objective symptoms (reported earlier in this article), we did not observe any significant and consistent changes in PR or QT/QT_c_ intervals, QRS complex, or ST segment (see Fig E5 in this article's Online Repository at www.jacionline.org). Two participants had evidence of a second-degree AV block type II at active challenge, but this did not cause any obvious cardiac symptoms, nor did it have any impact on cardiovascular measures.

With respect to HRV, we identified a number of significant changes at active challenge compared with the values at placebo challenge that were consistent with sympathetic activation: an increase in low-and high-frequency domains (*P* = .001 and *P* = .02 respectively [see Fig E6, *B* and *C* in this article's Online Repository at www.jacionline.org]) and a decrease in approximate entropy and sample entropy parameters (*P* = .009 and *P* = .001, respectively [see Fig E6, *D* and *E*]). However, these changes were not reproduced at repeat challenge (see Fig E7 in this article's Online Repository at www.jacionline.org). Interestingly, participants rated their level of anxiety as higher on a VAS at initial challenge than at repeat challenge (a median score of 4 [IQR = 1-6] at initial DBPCFC versus a median score of 3 [IQR = 1-5] at repeat challenge). Baseline challenges were associated with an increase in plasma epinephrine level (a median increase of 21.3% [IQR = –4.2%-47.2%] [*P* = .006]) compared with placebo, as a surrogate of endogenous catecholamine production (see Fig E2, *B*). Together, the changes in HRV, self-rated anxiety and plasma epinephrine level suggest significant anxiety and sympathetic drive at baseline challenge, which were not seen at repeat challenge and may therefore explain the increased CO at baseline but not at repeat challenge.

We did not observe any correlation between plasma epinephrine levels and HRV at initial DBPCFC (Spearman ρ < 0.25 for all HRV parameters).

### Impact of reaction severity on the changes in cardiovascular physiology

**Fig 5:**
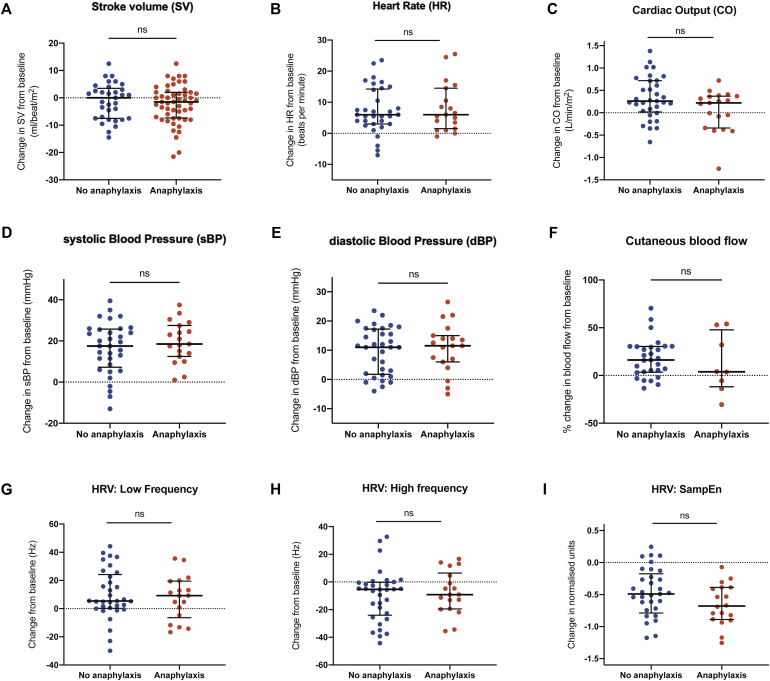
Changes in cardiovascular parameters at time of OCR from baseline, by reaction severity: SV(**A**), HR (**B**), CO(**C**), systolic and diastolic BP (**D** and **E**), cutaneous blood flow (**F**), low and high frequency domains in HRV (**G** and **H**), and sample entropy (SampEn) (**I**). Line and whiskers indicate medians and IQRs. Mann Whitney *U* test. *ns*, Not significant.

In all, 22 reactions (39%) met the international definitions for anaphylaxis, 9 of which were treated with intramuscular epinephrine according to local guidelines.^11^ A total of 12 subjects had a combination of cutaneous/mucosal and persistent gastrointestinal symptoms that met the international National Institute of Allergy and Infectious Disease/Food Allergy and Anaphylaxis Network definition for anaphylaxis,^8^ whereas 10 had objective laryngeal and/or lower respiratory symptoms. In 2 subjects, symptoms of anaphylaxis developed only after 2 or more hours following OCR (events that may have been triggered by consumption of a light meal); these reactions were therefore classified as nonanaphylaxis in this analysis. Surprisingly, there was no obvious impact of reaction severity, with similar changes in SV, HR, BP, blood flow, and HRV being seen at both anaphylaxis and milder reactions (Fig 5). Similarly, no difference was seen when anaphylaxis reactions treated with epinephrine (because of lower respiratory or severe laryngeal symptoms) were compared with those that were managed without epinephrine (see Fig E8 in this article's Online Repository at www.jacionline.org).

### Association between gastrointestinal symptoms and fluid redistribution

We compared cardiovascular parameters to subjects’ own rating of symptom severity using a 10-point VAS. Overall, there was a poor correlation between the change in cardiovascular parameters and participants’ VAS scores. However, a moderate correlation was seen between participants’ VAS scores for gastrointestinal symptoms and the change in SV and HR (see Table E1 in this article's Online Repository at www.jacionline.org). The moderate correlation between SV and VAS score for gastrointestinal symptoms was also seen at the repeat challenges (Spearman ρ = 0.40; *P* = .04).

**Fig 6:**
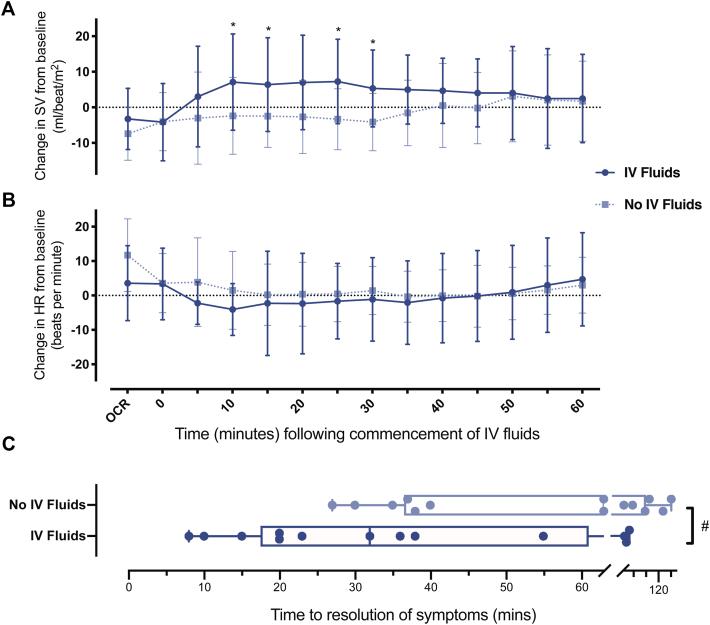
Impact of intravenous (IV) fluid administration following onset of OCR on the change in SV (**A**) and HR (**B**). Fourteen participants underwent a repeat challenge that resulted in similar symptom severity to their baseline challenge, and were treated with IV fluids (see text). SV and HR values are compared with the values at the equivalent time points for the initial DBPCFC, when no IV fluids were administered. Data are means ± SDs. ∗*P* < .05; *t* test. **C**, Time to resolution of gastrointestinal symptoms following OCR. Line and whiskers indicate medians and IQRs. ^#^*P* < .05; Wilcoxon signed rank test.

Interestingly, we observed anecdotally that administration of intravenous fluids following reaction was associated with apparent faster resolution of gastrointestinal cramping and pain. To study this further, 20 of the original participants underwent further peanut DBPCFC in a follow-up study (using a similar challenge protocol), in which abdominal pain was treated with intravenous fluids (500-1000 mL of 0.9% normal saline given over 30 minutes) following OCR. In all, 14 participants experienced a reaction similar to their baseline reaction to DBPCFC in terms of objective and subjective measures of organ involvement and symptom severity, including abdominal pain. This allowed a comparison of the changes in HR and SV between the original DBPCFC and subsequent challenge when intravenous fluids were administered to treat abdominal symptoms. Intravenous fluids resulted in a significant increase in SV from 10 minutes after administration compared with baseline DBPCFC, when no intravenous fluids were used (*P* = .04 [Fig 6]), reversing the decrease in SV observed at OCR. This was also associated with faster resolution of abdominal symptoms (*P* < .05 [see Table E2 in this article's Online Repository at www.jacionline.org]).

## Discussion

To our knowledge, this is the first study to undertake a detailed evaluation of cardiovascular events during food-related anaphylaxis and more mild reactions in humans. Our data suggest that significant cardiovascular changes occur during both mild and more severe peanut-induced allergic reactions, and this has important implications for clinical management. We observed a reproducible decrease in SV that was associated with increased peripheral blood flow; given the absence of significant peripheral edema during reactions, it is likely that the majority of fluid shifts occurred within the gut rather than into the cutaneous bed and thus contributed to the symptoms of abdominal discomfort reported during challenges. Significant fluid redistribution to the gut during anaphylaxis (including diffuse bowel wall edema confirmed on acute computed tomography scan) has been reported in a single case report of a patient with drug-induced anaphylaxis,^17^ but this has not yet been formally investigated in food allergy. There is growing evidence that the splanchnic circulation is an important reservoir of the venous circulation (eg, in chronic heart failure), and splanchnic nerve block alters venous return and cardiac preload.^18^ Our data suggest that gastrointestinal symptoms are associated with fluid redistribution, resulting in reduced venous return (cardiac preload). Alternatively, allergen-induced splanchnic venodilation may be another mechanism by which SV decreases during acute reactions.

We hypothesize that this decrease in cardiac preload drives a compensatory increase in peripheral vasoconstriction and HR, which maintains CO. The changes in HRV are consistent with sympathetic activation and similar to those reported in a pediatric population^19^; however, they were not reproduced at repeat food challenge. HRV has also been related to emotional arousal and responds to anxiety in a manner similar to that observed with sympathetic activation.^20^ The failure to reproduce the changes in HRV or CO may be related to the greater anxiety reported by participants at initial DBPCFC. Of note, SV would generally increase with sympathetic activation; thus, the decrease in SV is likely to be of even greater significance.

Remarkably, we observed no difference in the decrease in SV with severity: similar changes were seen for both anaphylaxis and milder reactions. This suggests that reduced preload is commonplace during peanut allergy reactions *irrespective of reaction severity*. Despite this, cardiovascular compromise is uncommon during food-induced anaphylaxis.^4^^,^^9^^,^^21^ Together, these observations imply that a major determinant of reaction severity may be the extent to which a host can compensate for the underlying reaction, which represents a new paradigm in understanding factors that contribute to severe outcomes in anaphylaxis.^4^ None of the anaphylaxis reactions in this study were refractory to treatment (no participant required more than 2 doses of intramuscular epinephrine), nor did we observe any significant symptoms of cardiovascular compromise. Patients experiencing anaphylactic shock (ie, anaphylaxis with significant circulatory compromise) are likely to have greater cardiovascular dysfunction, but it would not have been safe or ethical to attempt to evaluate hypotensive anaphylaxis in this study.

Existing data regarding the pathophysiology of food-induced anaphylaxis are limited and are mostly derived from animal models; moreover, results from animal models cannot always be extrapolated to humans.^21^ Vascular changes feature prominently in some animal models, with peripheral vasodilatation, venous pooling, and capillary leak in the splanchnic and hepatic circulation and/or vagal responses leading to reduced CO.^22^ Interstitial capillary leakage and vascular fluid extravasation have also been described.^23^^,^^24^ Data relating to human anaphylaxis are much more limited. Cardiovascular symptoms are less common with food-induced anaphylaxis than for anaphylaxis reactions triggered by non-food allergens. However, circulatory compromise appears to be important in more severe reactions, with a number of reports in the literature of fatal outcomes in food-induced anaphylaxis triggered by a change in posture,^5^^,^^6^ the putative mechanism being reduced venous return to the heart induced by the change in posture (for example, from lying/sitting to standing position) in the context of peripheral vasodilatation due to the allergic reaction.

In a landmark article, Fisher described 205 patients with perioperative anaphylaxis, many of whom had central venous monitoring *in situ*.^25^ He reported low right-filling pressures in all patients without cardiac disease. Furthermore, by monitoring serial changes in hematocrit during the reactions in 22 patients, he concluded that anaphylaxis can cause a loss of circulating volume of up to 35% within 10 minutes of reaction. These data lend weight to the possibility that poor outcomes triggered by postural changes during anaphylaxis are due to a failure to compensate for fluid redistribution, with consequent loss of CO. Preexisting cardiovascular disease is a risk factor for severe anaphylaxis^26^; the changes seen in this study may be more significant in those with cardiovascular disease. Our data suggest that acute coronary syndrome as a consequence of anaphylaxis (Kounis syndrome) or arrhythmias are uncommon in this setting of food-induced allergic reactions of mild-to-moderate severity in otherwise healthy volunteers.

Management of anaphylaxis should involve the early administration of intramuscular epinephrine.^27^ Since 2011, the guidelines have also included the recommendation that the affected individual lie in a supine position with the legs raised to optimize venous return (unless this compromises breathing).^27^ Our data provide an evidence base not only for postural support but also for the administration of intravenous fluids, which is currently recommended only for treating hypotension or circulatory shock,^11^^,^^28^ both of which are late, preterminal signs in children and young adults, the age groups that are at greatest risk of fatalities due to food-induced anaphylaxis. In this study, administration of 500 to 1000 mL of normal saline restored SV and resulted in faster resolution of abdominal symptoms than treatment without fluids did. This suggests that abdominal pain during anaphylaxis may be linked to visceral angioedema, which can be reversed with rapid fluid administration. We therefore propose that appropriate fluid resuscitation be considered early in cases of food-induced anaphylaxis that are refractory to initial treatment with epinephrine—at least in those *without* preexisting cardiovascular disease—to maintain adequate circulatory volume and aid the distribution of the administered epinephrine.

### Limitations

Anaphylaxis research is challenging, primarily because of the risks inherent in inducing potentially life-threatening reactions. Indeed, the evidence for epinephrine as first-line treatment is derived from case series, animal studies, and expert opinion.^29^ To our knowledge, this is the first systematic study of cardiovascular changes during food-induced allergic reactions, including anaphylaxis. Although 39% of reactions evaluated met the international clinical criteria for anaphylaxis, only 9 (16%) resulted in objective lower respiratory symptoms, and we did not observe any episodes of refractory anaphylaxis or hypotension, which limits the inferences which can be made. Caution is needed when interpreting these data because this was a challenge study and thus is not necessarily representative of peanut-induced allergic reactions occurring in the community. We found evidence that some of the changes seen (such as increased HR and BP) might have been exaggerated by a sympathetic response due to anxiety in study participants. As we have already noted, however, this does not explain the observed decrease in SV, something which was observed at repeat challenge. Whether the other changes seen might have been reproduced had more participants agreed to a further DBPCFC is unclear.

Our findings may also have been limited by the size of the study cohort; all of our participants were relatively healthy adults, mostly with a history of nonsevere reactions. Preexisting cardiovascular disease has been shown to be a risk factor for fatal anaphylaxis; it is therefore possible that different changes may be seen in those with preexisting cardiovascular disease.

Of note, in a case series of 7 patients receiving intramuscular epinephrine for anaphylaxis and monitored by using identical equipment, we observed no significant changes in cardiovascular parameters (including SV) other than for a minor increase in HR.^30^ Therefore, the size of the effect of a single dose of intramuscularly administered epinephrine on cardiovascular parameters during food-triggered anaphylaxis remains unclear, although published case series clearly demonstrate an advantage in administering both intravenous fluids and adrenaline together to treat severe reactions.^31^ Clearly, the majority of anaphylaxis reactions do respond to treatment with epinephrine, without the need for fluid resuscitation; for this reason, we recommend that appropriate fluid resuscitation be considered early in cases of food anaphylaxis that are refractory to initial treatment with epinephrine, or in cases in which significant abdominal pain continues to persist despite resolution of other symptoms.

### Conclusions

Significant changes in cardiovascular function, including decreased SV, occur during peanut-induced allergic reactions in adults, irrespective of severity. These data provide a sound rationale for optimizing venous return during anaphylaxis through postural maneuvers and fluid resuscitation in the management of refractory reactions, even in the absence of apparent cardiovascular compromise.

**Clinical implications**

Significant changes in cardiovascular function, including decreased stroke volume, occur during peanut-induced allergic reactions in adults irrespective of severity; these changes (and associated symptoms such as abdominal pain) can be reversed through early fluid resuscitation.

## Supplementary data

Fig E1

Fig E2

Fig E3

Fig E4

Fig E5

Fig E6

Fig E7

Fig E8

Tables E1-E2
