# Supplementary figures and images for: Cardiovascular changes during peanut-induced allergic reactions in human subjects
Source: J Allergy Clin Immunol. 2021 Feb;147(2):633–42. doi: 10.1016/j.jaci.2020.06.033 (PMC7858218; doi:10.1016/j.jaci.2020.06.033)

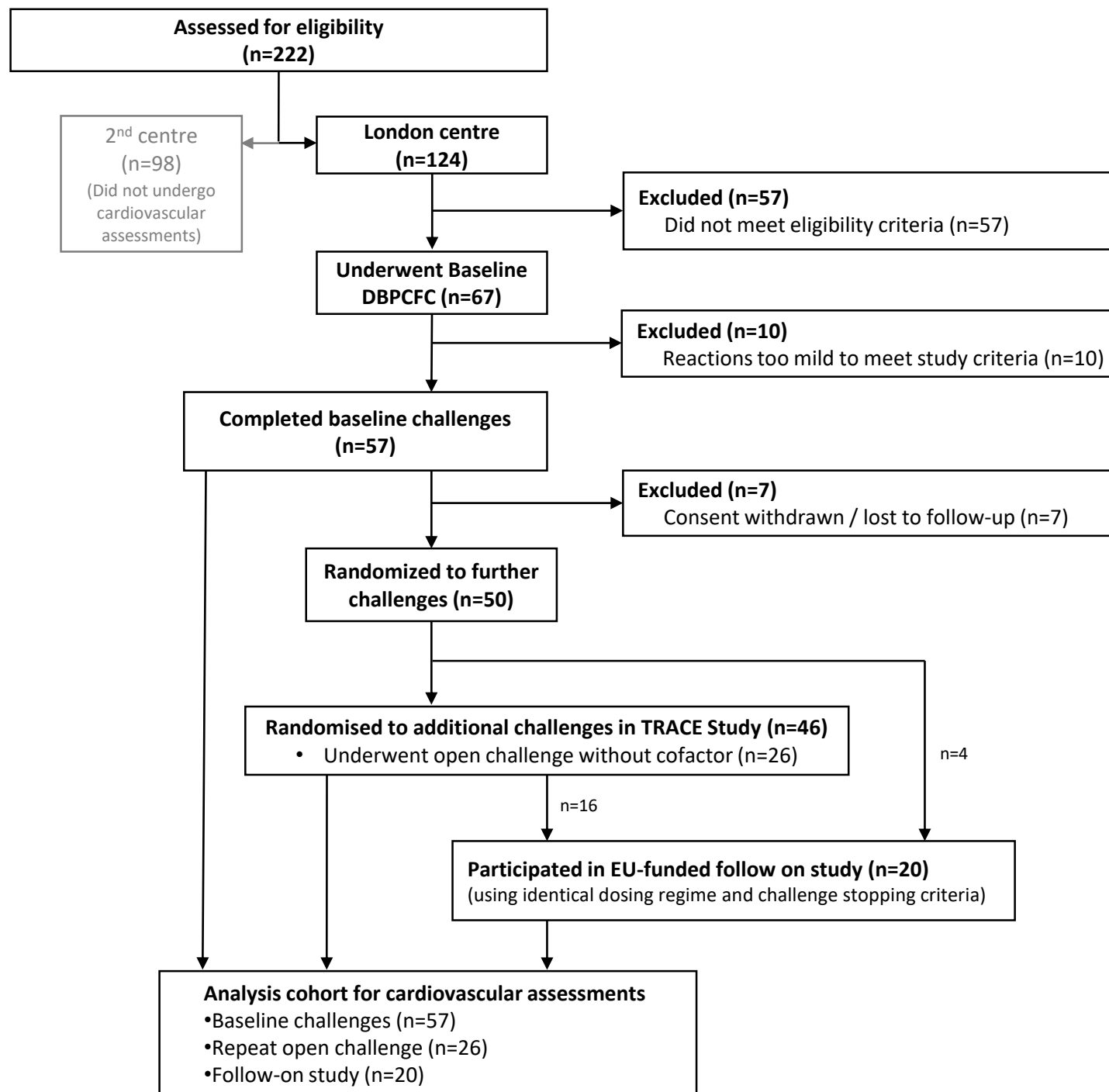

Supplement: Fig E1 [file mmc1.pdf]
