# Supplementary material for: Cardiovascular changes during peanut-induced allergic reactions in human subjects
Source: J Allergy Clin Immunol. 2021 Feb;147(2):633–42. doi: 10.1016/j.jaci.2020.06.033 (PMC7858218; doi:10.1016/j.jaci.2020.06.033)
Supplement: Fig E2 [file mmc2.pdf]

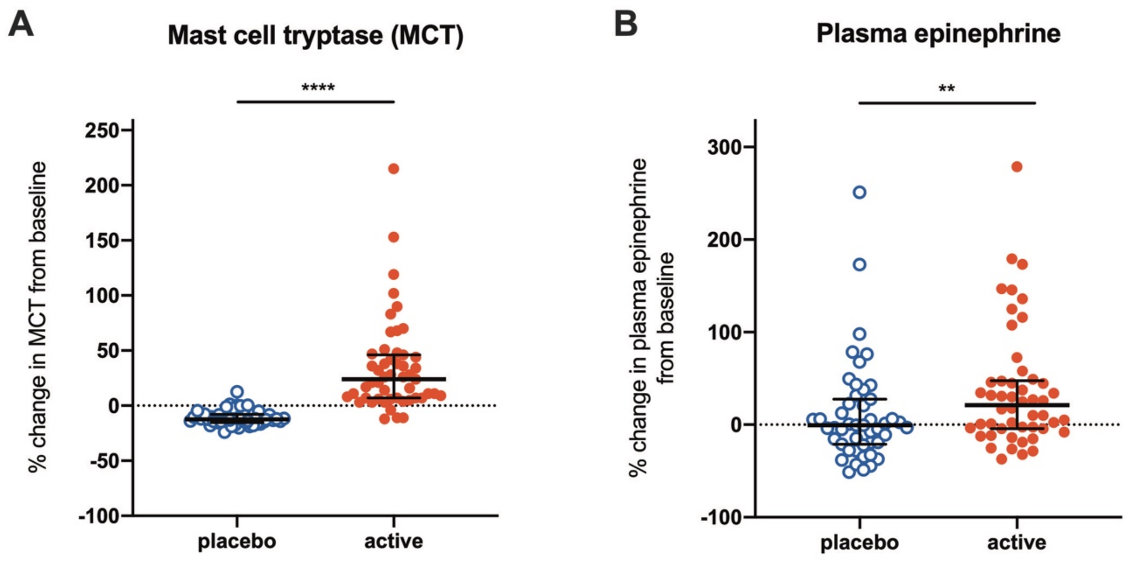

Figure E2. (**A**) Peak MCT increased significantly compared to baseline at active challenge (p<0·001, Wilcoxon SR test) and compared to the change in MCT at the placebo challenge (p<0·001, Wilcoxon SR test), implying systemic mast cell degranulation. (**B**) A significant increase was also seen in plasma epinephrine levels compared to baseline as a marker of endogenous catecholamine production (collected prior to treatment of reaction) at active challenge (p<0·001, Wilcoxon SR test), and compared to the change at placebo challenge (p=0·006, Wilcoxon SR test). Line and whiskers indicate median and IQR. ****p<0·0001; **p<0·01, Wilcoxon SR test.
