# Supplementary material for: Cardiovascular changes during peanut-induced allergic reactions in human subjects
Source: J Allergy Clin Immunol. 2021 Feb;147(2):633–42. doi: 10.1016/j.jaci.2020.06.033 (PMC7858218; doi:10.1016/j.jaci.2020.06.033)
Supplement: Fig E3 [file mmc3.pdf]

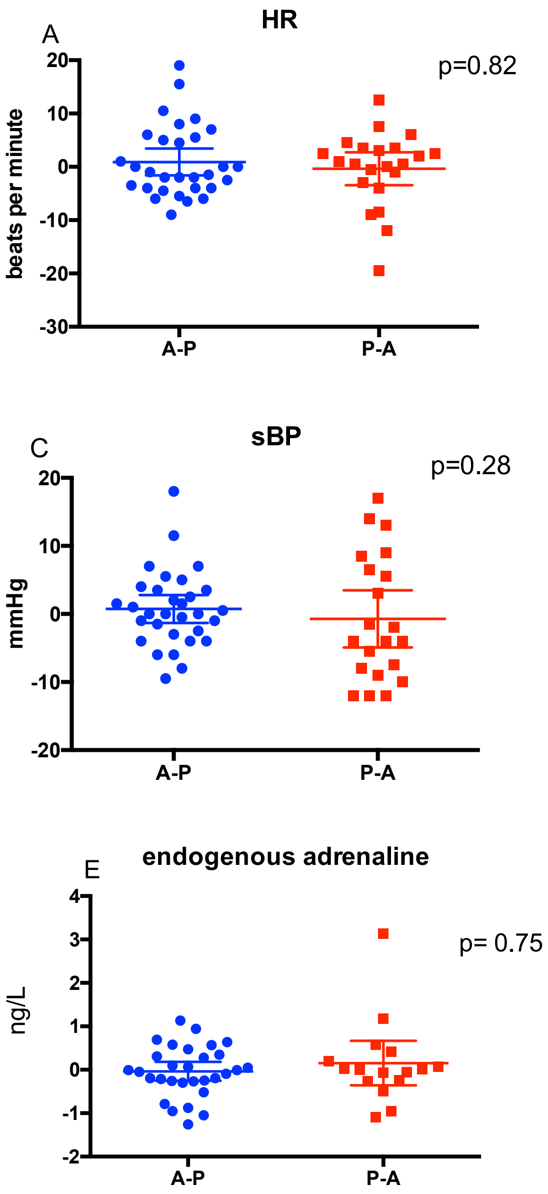

Figure E3. Difference in HR, SV, BP and plasma epinephrine levels compared to baseline, by order of challenges at DBPCFC. Data shown refer to the placebo challenge occasion. A-P corresponds to those participants having placebo second, P-A corresponds to those participants having placebo as the first challenge intervention.
