# Supplementary material for: Cardiovascular changes during peanut-induced allergic reactions in human subjects
Source: J Allergy Clin Immunol. 2021 Feb;147(2):633–42. doi: 10.1016/j.jaci.2020.06.033 (PMC7858218; doi:10.1016/j.jaci.2020.06.033)
Supplement: Fig E4 [file mmc4.pdf]

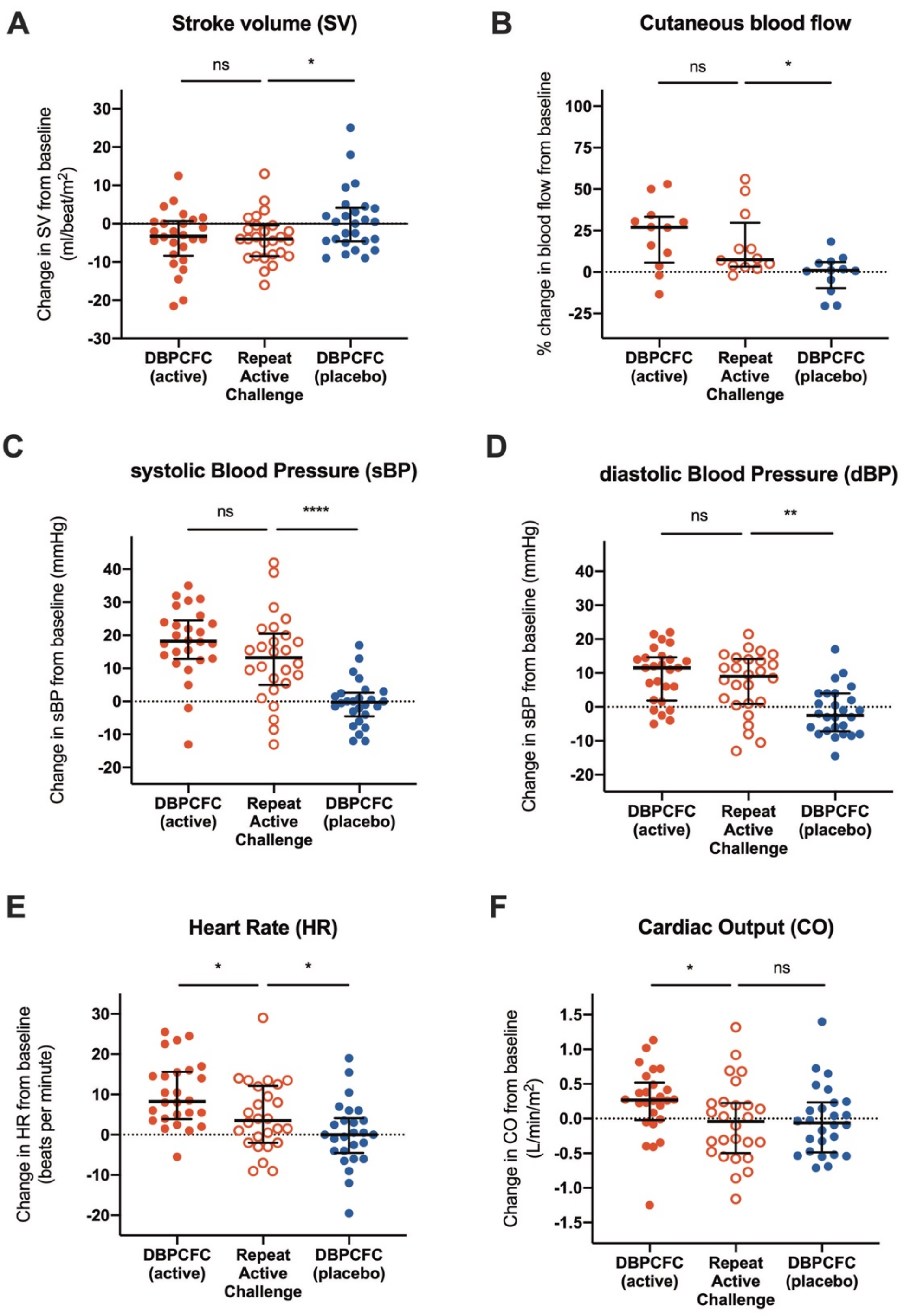

Figure E4. Changes in cardiovascular parameters at time of objective clinical reaction (OCR) during peanut-induced allergic reactions at further open peanut challenge in 26 individuals. (**A**) Stroke volume, (**B**) Cutaneous blood flow, (**C** and **D**) systolic and diastolic blood pressure, (**E**) heart rate and (**F**) cardiac output. Line and whiskers indicate median and IQR. ****p<0·0001; **p<0·01; *p< 0·05, Wilcoxon SR test.
