# Supplementary material for: Cardiovascular changes during peanut-induced allergic reactions in human subjects
Source: J Allergy Clin Immunol. 2021 Feb;147(2):633–42. doi: 10.1016/j.jaci.2020.06.033 (PMC7858218; doi:10.1016/j.jaci.2020.06.033)
Supplement: Fig E5 [file mmc5.pdf]

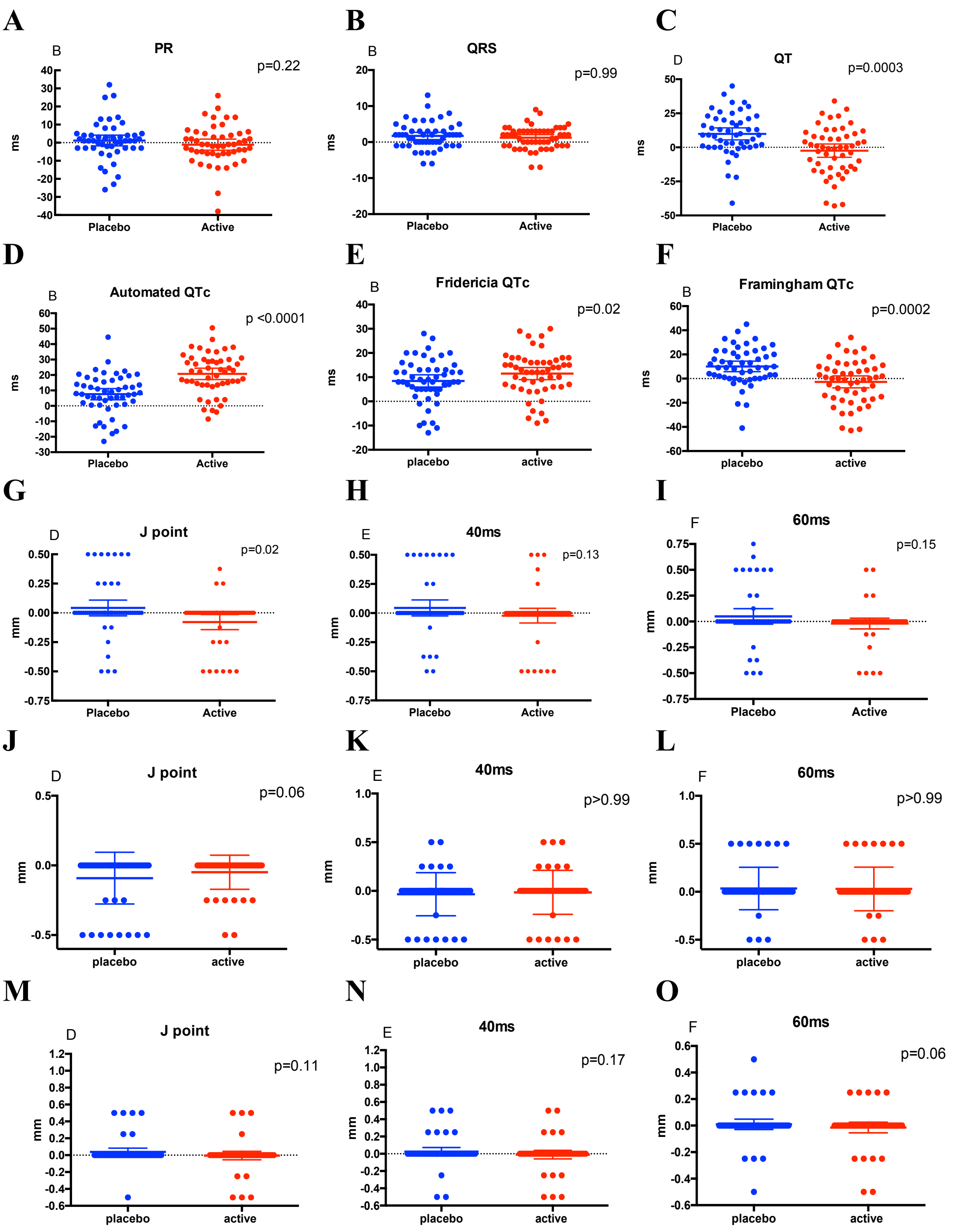


**Figure E5**. Changes in ECG parameters at time of objective clinical reaction (OCR) during peanut-induced allergic reactions compared to placebo challenge. (**A**) PR interval, (**B**) QRS interval, (**C-F**) QT/QTc interval, (**G-I**) ST interval on lead II at (**G**) J point, (**H**) 40ms and (**I**) 60ms; (**J-L**) on lead V2 at (**J**) J point, (**K**) 40ms and (**L**) 60ms; (**M-O**) ST interval on lead V6 at (**M**) J point, (**N**) 40ms and (**O**) 60ms.
