# Supplementary material for: Cardiovascular changes during peanut-induced allergic reactions in human subjects
Source: J Allergy Clin Immunol. 2021 Feb;147(2):633–42. doi: 10.1016/j.jaci.2020.06.033 (PMC7858218; doi:10.1016/j.jaci.2020.06.033)
Supplement: Fig E6 [file mmc6.pdf]

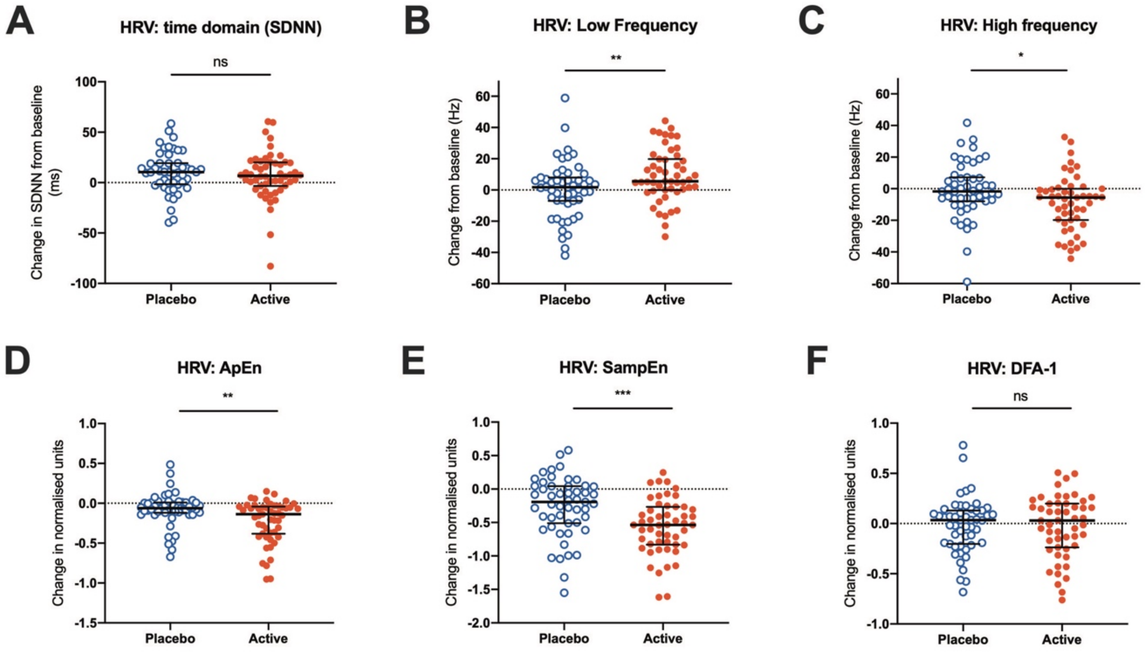

Figure E6. Changes in heart rate variability (HRV) parameters at time of objective clinical reaction (OCR) from baseline during peanut-induced allergic reactions at active challenge compared to placebo. (**A**) standard deviation of the normal-normal RR interval (SDNN); (**B** and **C**) low and high frequency domains; (**D** to **F**) non-linear methods: (**D**) approximate entropy (ApEn); (**E**) sample entropy (SampEn); (**F**) short-term fractal scaling exponent (DFA-1). Line and whiskers indicate median and IQR. ***p<0·001; **p<0·01; *p<0·05, Wilcoxon SR test.
