# Supplementary material for: Cardiovascular changes during peanut-induced allergic reactions in human subjects
Source: J Allergy Clin Immunol. 2021 Feb;147(2):633–42. doi: 10.1016/j.jaci.2020.06.033 (PMC7858218; doi:10.1016/j.jaci.2020.06.033)
Supplement: Fig E7 [file mmc7.pdf]

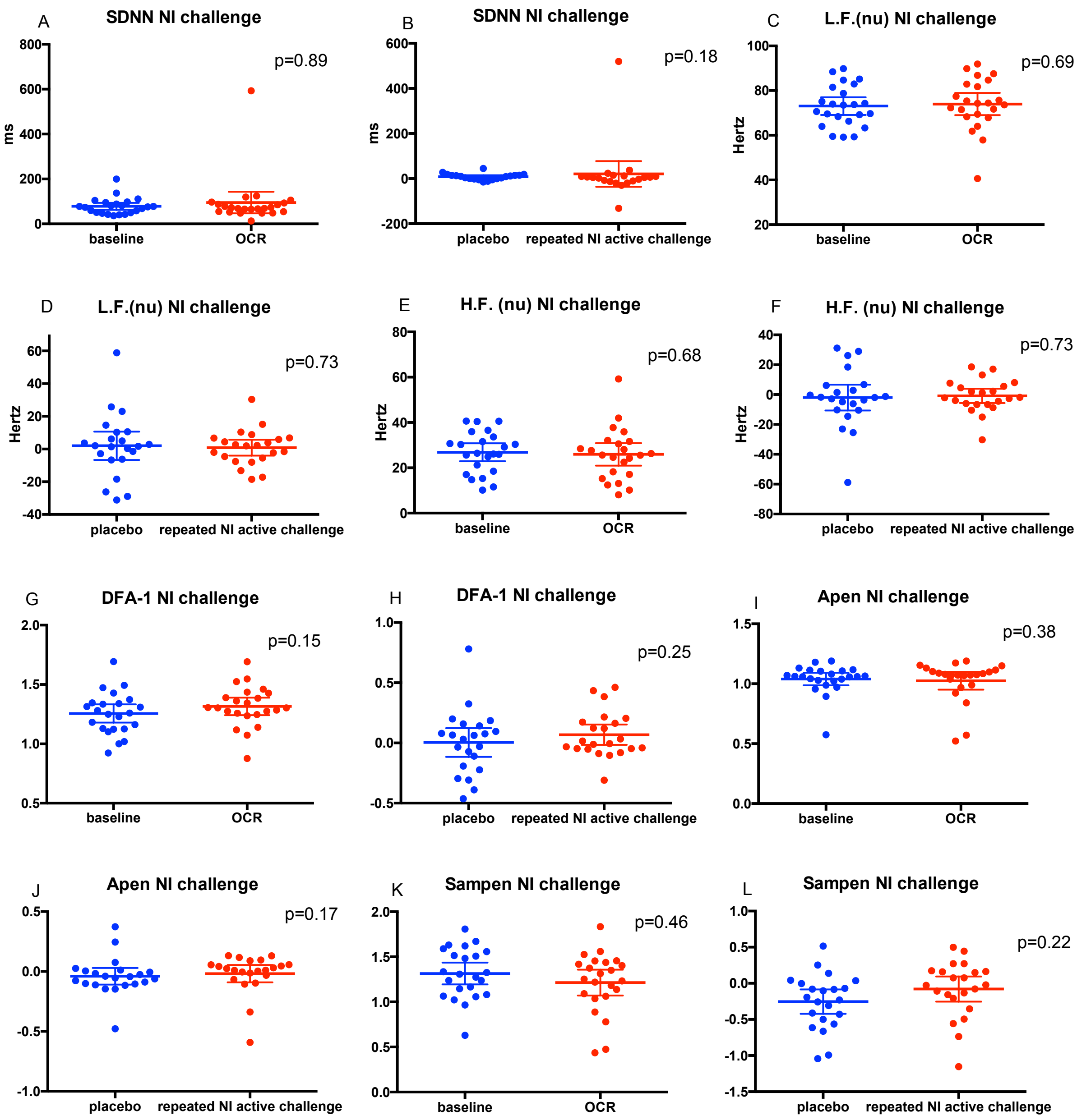

Figure E7. Changes in heart rate variability (HRV) at time of objective clinical reaction (OCR) during peanut-induced allergic reactions at repeat open challenge. (**A**) SDNN, (**C** and **E**) low and high frequency domains, (**G**) DFA-1, (**I**) approximate entropy (ApEn) and (**K**) sample entropy (SampEn); and at active repeat challenge compared to placebo for (**B**) SDNN, (**D**) LF, (**F**) HF, (**H**) DFA-1, (**J**) Apen and (**L**) Sampen. All P values, Wilcoxon SR test.
