# Supplementary material for: Cardiovascular changes during peanut-induced allergic reactions in human subjects
Source: J Allergy Clin Immunol. 2021 Feb;147(2):633–42. doi: 10.1016/j.jaci.2020.06.033 (PMC7858218; doi:10.1016/j.jaci.2020.06.033)
Supplement: Fig E8 [file mmc8.pdf]

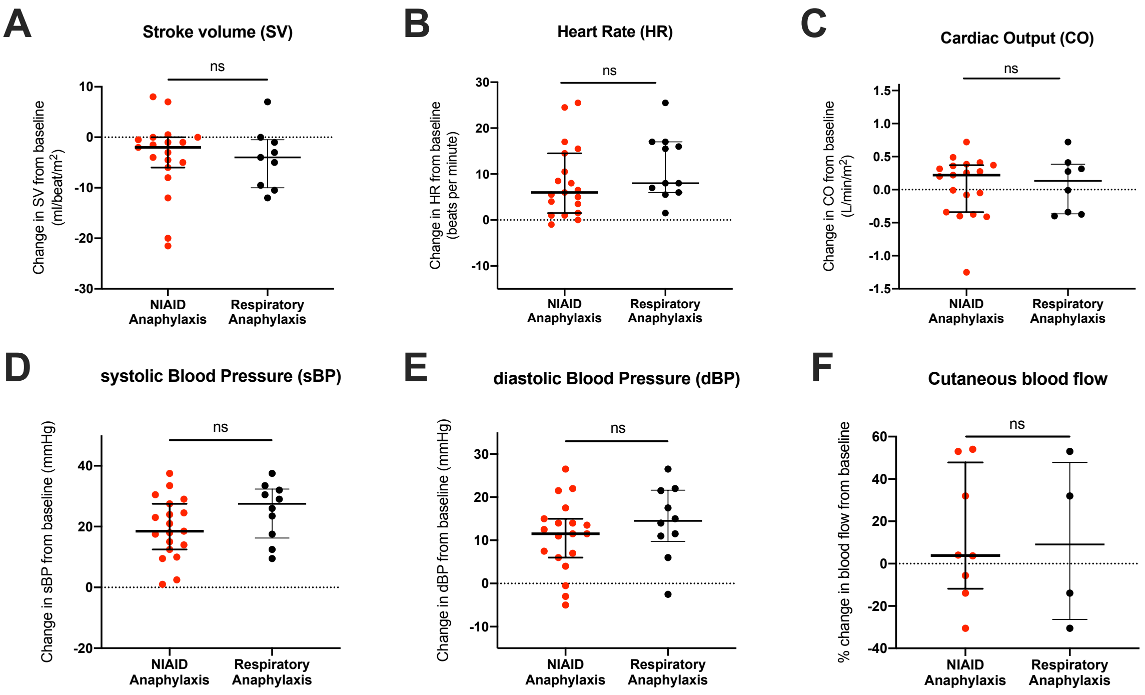

Figure E8. Changes in cardiovascular parameters at time of objective clinical reaction (OCR) from baseline, by anaphylaxis severity. (**A**) Stroke volume, (**B**) heart rate, (**C**) cardiac output, (**D** and **E**) systolic and diastolic blood pressure, (**F**) Cutaneous blood flow. Line and whiskers indicate median and IQR. ns, not significant, Mann Whitney U-test.
