# Supplementary material for: Cardiovascular changes during peanut-induced allergic reactions in human subjects
Source: J Allergy Clin Immunol. 2021 Feb;147(2):633–42. doi: 10.1016/j.jaci.2020.06.033 (PMC7858218; doi:10.1016/j.jaci.2020.06.033)
Supplement: Tables E1-E2 [file mmc9.pdf]

SUPPLEMENTARY TABLES

Table E1. Correlation between participants´ VAS score to rate severity of symptoms by organ, and CVS parameters.

	**Visual Analogue Score (VAS)**			
	Skin	Gastrointestinal	Upper respiratory	Lower respiratory
**SV**	r = 0·21 (p=0·14)	**r = -0·43 (p=0·002)**	r = 0·08 (p=0·6)	r = -0·31 (p=0·03)
**HR**	r = 0·08 (p=0·56)	r = 0·30 (p=0·03)	r = 0·12 (p=0·43)	r = 0·11 (p=0·47)
**sBP**	r = 0·03 (p=0·83)	r = 0·20 (p=0·18)	r = 0·22 (p=0·24)	r = 0·14 (p=0·35)
**dBP**	r = -0·17 (p=0·24)	r = 0·22 (p=0·14)	r = 0·27 (p=0·06)	r = -0·02 (p=0·88)
**Blood flow**	r = -0·10 (p=0·57)	r = 0·15 (p=0·39)	r = -0·06 (p=0·74)	r = -0·30 (p=0·07)

Spearman's R. Highlighted, p<0·01

Table E2. Comparison of reaction severity in 14 patients who experienced gastrointestinal symptoms at baseline DBPCFC, and similar symptoms at a subsequent challenge in a follow-on study treated with IV fluids.

	Initial challenge (no IV fluids)	Subsequent challenge (IV fluids administered)	P value	Cohen's Kappa
Eliciting dose (mg peanut protein)	133 (33 to 433)	83 (26 to 433)	0·97	0·50
Symptom grade	3 (2 to 3)	3 (2 to 4)	0·35	0·53
Patient's assessment of abdominal pain	6.5 (4 to 8)	6 (4 to 7)	0·60	0·48
% increase in MCT (from baseline)	13 (0 to 28)	20 (2 to 40)	0·64	-
Time to symptom resolution (mins)	63 (37 to 102)	32 (18 to 61)	**0·02**	-

Data are median (IQR).
